# Physical Fighting among School-Attending Adolescents in El Salvador: An Examination of the 2013 Global School-Based Health Survey

**DOI:** 10.3390/ijerph17041248

**Published:** 2020-02-15

**Authors:** Mazin Omer, Masood Ali Shaikh, Mariella Stiller, Michael Lowery Wilson

**Affiliations:** 1 Injury Epidemiology and Prevention Research Unit, Heidelberg Institute of Global Health (HIGH), University of Heidelberg, 69120 Heidelberg, Germany; mazin1255@gmail.com (M.O.); mariella.stiller@gmx.de (M.S.); 2 Injury Epidemiology and Prevention (IEP) Research Group, Turku Brain Injury Centre, Turku University Hospital and University of Turku, 20520 Turku, Finland; masoodalishaikh@gmail.com

**Keywords:** school health, interpersonal violence, mental health, injury prevention, epidemiology

## Abstract

**Background:** Violence among school-attending adolescents is an important public health problem worldwide. The present study examined demographic correlates for physical fighting behavior among a nationally representative sample of school-attending adolescents in El Salvador. **Methods:** Initial cross-tabulations to screen for correlations was then followed by logistic regression to understand the direction and the strength of selected demographic variables for physical fighting behavior, which occurred within a 12 month period of recall. **Results:** Out of a sample of 1910 school-attending adolescents in El Salvador, 11.5% reported having been involved in two or more physical fights during the recall period. Regression analyses indicated that being male (OR = 3.55; 95% CI = 2.11–6.00); having experienced bullying (OR = 2.16; 95% CI = 1.44–3.24); physical activity (OR 0.61; 95% CI 0.46–0.80); a sedentary lifestyle (OR 1.54; 95% CI 1.05–2.27), suicide planning (OR 2.28; 95% CI 1.46–3.56), and having non-understanding parents (OR = 1.45; 95% CI 1.06–1.98) were significantly associated with physical fighting among the sampled adolescents. **Conclusion:** Within the limitations of cross-sectional surveys conducted in school settings, the results of the present study suggest that giving attention to preventing bullying behavior among males and involving parents should be components of a multi-pronged strategy to preventing physical fighting in schools in El Salvador.

## 1. Introduction

Violence is defined as the intentional use of physical force against another person that may result in injury or psychological harm [1]. Violence among adolescents is considered a major public health problem and is associated with serious physical and psychosocial consequences [2,3]. Some of the psychological consequences of school violence include post-traumatic stress as well as depressive symptoms [4,5]. Those who experience violence in school settings, in particular, may suffer both academically and socially [6].

Violence in schools has a negative impact on the school environment by creating an atmosphere of anxiety, fear, and insecurity. It also acts as an impediment to the rights of students, including their right to an education [7]. Although violence cuts across all socio-economic groups, it is more prevalent among males [8,9] and lower socio-economic settings, including low- and middle-income countries [10,11]. Multiple factors have been found to be correlated with violence among young people. Some authors have argued that violence goes hand in hand with psychosocial stress, with poverty status playing a role in increasing stress levels [12,13]. Moreover, psychological factors such as loneliness, friendship, and parental support have been potentially linked to violent behavior among adolescents [14,15]. Physical inactivity as well as a sedentary lifestyle have also been found to be associated with violence among adolescents in schools [16,17].

Adolescents differ cognitively and developmentally from adults, but also in terms of their primary social environments. Exploring the role that factors within these environments have on the expression of interpersonal violence is necessary to understand avenues for preventive strategies [18]. Recent reports have shown that as a result of violence, thousands of children are out of school in El Salvador, where almost one in three children drop out of school before completing secondary school [19]. In the present study, we examined a number of risk factors related to physical fights, as one manifestation of violence, among adolescents in schools in El Salvador. The goal of this study was to examine the prevalence and correlates for physical fighting among school-attending adolescents in El Salvador.

## 2. Methods

### 2.1. Data Description

The data for this study was derived from the Republic of El Salvador contribution to the 2013 Global School-based Health Survey (GSHS). The methodology for the GSHS was developed collaboratively by the World Health Organization and the United States Centers for Disease Control and Prevention. All data used in this study are freely available and accessible via the WHO website [20]. A two-stage cluster sampling design was used to produce representative data of all students enrolled in grades 7 to 9 in the country.The survey was administered to school-attending adolescents in grades 7, 8, and 9—usually attended by aged 13 to 16 years old. Self-reported information on indices pertaining to health-risk behaviors was collected. In El Salvador, 1915 students aged 11 years or younger to 16 years or older (weighted percentage: 48.5% female) completed the questionnaire. The overall response rate was 88%, with 100% of schools responding to the survey. The analysis presented in this paper did not exclude any cases; 32 records missing gender and 29 records missing age (11 records missing both) were included in the analysis to correctly analyze the sampling design used.

### 2.2. Setting

This study is based on data collected in the Republic of El Salvador, a Central American country bordering the Pacific Ocean in the South, Guatemala in the Northwest, and Honduras in the Northeast. El Salvador had a population of approximately 6.34 million inhabitants in 2016. The country’s gross national income per capita lies between 996 and 3895 US$, and as such, the country is classified as a lower-middle income country [21].

### 2.3. Measurements

The dependent variable “physical fighting” was derived from the survey question “During the past 12 months, how many times were you in a physical fight?” Response options ranged were: “0 times”, “1 time”, “2 or 3 times”, “4 or 5 times”, “6 or 7 times”, “8 or 9 times”, “10 or 11 times”, and “12 or more times”. For the purpose of our analyses, participants were classified as having participated in a physical fight if they reported being in two or more fights (N = 238), in order to identify problematic fighting behavior. If none or one fight was reported, participants were classified as not participating in a physical fight (N = 1672); for five records, this information was missing.

We investigated thirteen independent variables to screen for statistically significant associations with the dependent variable—nine at the individual level (age, sex, anxiety, loneliness, bullying victimization, truancy, physical inactivity, sedentary lifestyle, and suicide planning) and four independent variables at the social level (food insecurity, number of close friends, understanding parents, and unhelpful peers). All independent variables apart from age and number of close friends were dichotomized according to the distribution of the data in order to facilitate analysis (Table 1).

### 2.4. Statistical Analysis

Because only eight students who were either 11 years old or younger participated in the survey, they were combined with the next age category of 12 years for analysis. Univariate analyses characterized the distribution of each selected variable among those who were either never or only once involved in a physical fight versus those who were involved two or more times. This was followed by bivariate analyses utilizing associations among students who were either never or only once involved in a physical fight versus those who reported involvement in two or more physical fights. The categorical variables were explored with survey versions of a chi-square test and continuous variables with a *t*-test.

All independent variables found to be statistically significant at a *p*-value of <0.05 in the bivariate analysis were included in the multivariate analysis by using the design-based multiple logistic regression model. Odds ratios (OR), including their 95% and 99% confidence intervals, are reported for the strength and direction of associations between involvement in physical fights and the factors studied. Stata version 15 (StataCorp LP, 2017) was used for the data analysis.

## 3. Results

Within the recall period of 12 months, 11.5% (unweighted count: 238) of the participants reported being involved in two or more physical fights. The mean age of the sample was 14.2 years (SD: 1.1) and more than half (51.5%) was male. Bullying victimization was reported by 22.6% of the respondents. Only 74.9% reported not being physically active for a total of at least 60 min per day in the past seven days, for three days or fewer. A sedentary lifestyle was defined as spending three or more hours during a typical or usual day sitting and watching television, playing computer games, talking with friends, or doing other seated activities, such as chatting, playing on a cell phone, talking on phone, or using an iPad or tablet, and was reported by 34.2%. During the past 12 months, 11.4% reported having made a plan about how they would attempt suicide. Almost half of the participants (50.2%) reported that their parents or guardians either never, rarely, or sometimes understood their problems and worries during the previous 30 days. Moreover, 9.0% felt lonely most of the time or always in the previous 12 months.

### 3.1. Bivariate Analysis

The bivariate analyses show that within all of the selected variables with the exception of age and food insecurity, statistically significant differences existed between students who had been involved in physical fights and those who had not (Table 2). There was no significant difference in the mean age of students who were involved in physical fights when compared with those who were not (14.4% and 14.2%, respectively, *p*-value: 0.16). The proportion of food insecurity in students who were involved in physical fights compared with those who were not was also not significant (5.7% and 3.2%, respectively, *p*-value: 0.10). In the bivariate analyses, the majority of adolescents reporting involvement in 2 or more physical fights were male (70.9%). Furthermore, among Salvadorian adolescents who were involved in physical fights, 40.5% were victims of bullying, almost two thirds were physically inactive (63.6%) and reported having non-understanding parents (63.4%), 13.8% were anxious, and 12.8% were lonely. More than one fifth (22.8%) had a plan to attempt suicide, 16.6% reported truancy, 58.6% had unhelpful peers, and the mean number of close friends was 2.4 (SD: 1.03).

### 3.2. Multivariate Analyses

An age- and sex-adjusted analysis (Table 3) was conducted for all 13 variables used. The analyses revealed statistically significant associations for all selected variables, with the exception of truancy, age, and food insecurity. Males were 2.50 times more likely to have been involved in physical fights in the past 12 months than females (95% CI: 1.60–3.85). Adolescents who experienced bullying were 2.82 times more likely to have been involved in physical fights than those whonever experienced bullying (95% CI: 2.05–3.88). Being physically inactive was associated with decreased involvement in physical fights (OR = 0.60; 95% CI: 0.46–0.79). A sedentary lifestyle (OR = 1.91; 95% CI: 1.30–2.80) and having made suicide plans (OR = 3.40; 95% CI: 2.10–5.49) were positively associated with physical fighting behavior. Students who reported having felt lonely (OR = 2.10; 95% CI: 1.37–3.23) and anxious (OR = 2.96; 95% CI: 1.88–4.67) were more likely to have been involved in physical fights. Not having understanding parents (OR = 1.97; 95% CI: 1.54–2.52) and having unhelpful peers (OR = 1.71; 95% CI: 1.22–2.39) were associated with higher reports of being involved in physical fights. Having close friends (OR = 0.98; 95% CI: 0.80–0.99) was associated with fewer reports of physical fights. All associations found to be statistically significant at a <0.05 *p*-value were also found to be statistically significant at a <0.01 *p*-value, with the exception of close friends. .

The final model was adjusted for all associated covariates found to be statistically significant—using a *p*-value of <0.05—in the bivariate analysis (Table 4). Adolescents who are male (OR 3.55; 95% CI 2.11–6.00), victims of bullying (OR 2.16; 95% CI 1.44–3.24), sedentary (OR 1.54; 95% CI 1.05–2.27), have non-understanding parents (OR 1.45; 95% CI 1.06–1.98), and have planned to attempt suicide (OR 2.28; 95% CI 1.46–3.56) were more likely to be involved in physical fights in the past 12 months. Being physically inactive (OR 0.61; 95% CI 0.46–0.80) was significantly associated with fewer reports of involvement in physical fighting. All of these variables were also statistically significant using the 99% CI, i.e., at a *p*-value of <0.01, with the exception of reporting a sedentary lifestyle (99% CI: 0.91–2.61) and having non-understanding parents (99% CI: 0.94–2.22). The goodness-of-fit test revealed that this multivariate logistic model was a good fit for physical fighting among El Salvadoran school students (F(9,13): 0.56, *p*-value: 0.805).

## 4. Discussion

The goal of this study was to examine the prevalence and correlates of physical fighting among school-attending adolescents in El Salvador. The study revealed that 11.5% of participants were involved in a physical fight in the 12 months prior to being surveyed. In various high-income countries, high rates of physical fighting have been reported among similarly aged school-based samples: a study conducted in North America and Europe presented an overall average of physical fighting of 58% [22], which is consistent with rates of physical fighting behavior among high school students in the USA [23]. Moreover, a study conducted in Santiago, Chile, aa high-income South American country, showed higher rates (40.7%) of physical fighting among school-attending adolescents [24]. Low-income countries like Namibia and Ghana, using a lower threshold for physical fighting behavior, report relatively high rates of fighting behavior among adolescents: 50.6% and 32%, respectively [25,26].

Consistent with the literature, we found a significant association between gender and physical fighting among adolescents, with males being over-represented [27,28]. In comparison, the gender gap was smaller in the previous study conducted in Namibia (55.2% and 46.2% among boys and girls, respectively) [25]. In high-income countries, significant differences by gender in physical fighting among adolescents exists, with the highest prevalence being reported in eastern and central Europe [22]. This gender gap can reflect cultural norms in settings where males may be encouraged to engage in displays of power and aggressive behavior [27]. Research has suggested that traditional masculine gender socialization and social norm models encourage males to engage in behaviors that put their health at risk [29]. In addition, the developmental onset of some hormones is correlated with the onset of aggresivity and violent behavior in adolescent boys [30], which may also partially explain the phenomena. Furthermore, studies have shown that the type of violence differs by gender. Males tend to be involved in physically aggressive actions, such as physical fighting, while females are more likely to be verbally aggressive and engage in indirect forms of violence [25].

Participants in the study who engaged in physical fighting were more likely to be victims of bullying and to be physically attacked by peers. This is consistent with previously conducted research [31,32]. This finding strengthens the idea that physical fighting is related to other negative peer behavior characteristics among adolescents [26]. Victims of bullying are more likely to be engaged in physical fighting, potentially as a way of defending themselves [16]. Adolescents who previously had been victims of violence are also likely to carry weapons in schools; thus potentially contributing to further serious consequences that may arise during altercations [2,24].

Consistently, the study revealed that students with sedentary lifestyles were more likely to have been involved in physical fighting. The latter was examined in some studies in which sedentary behavior was predictive of violence-related behavior [33,34]. Higher crime rates in society overall have also been found to be associated with higher levels of media usage [35]. Adolescents who primarily spend their free time watching television and videos were more likely to engage in other negative risk behaviors [36]. Moreover, a study conducted among young Japanese students found an association between playing certain video games and aggression [37]. In contrast to the literature, we found that physically active students were more likely to be involved in physical fights. A study that examined this association in 40 countries in Europe and North America revealed that frequent screen-based sedentary activities, overweight/obesity, and physical inactivity were significantly associated with higher rates of physical fighting among adolescents [17]. Nevertheless, participation in a range of physical-activity-related behaviors is associated with less involvement in other risk behaviors (e.g., alcohol and marijuana intake) [36] and hence, less physical fighting [23]. However, more research is needed to examine the relationship between physical activity, a sedentary lifestyle, and their determinants, and physical fighting in low- and middle-income countries.

Our study revealed an increased risk of involvement in physical fights among adolescents who had made a plan to commit suicide. Congruent with this finding, a study in China documented an association between suicide and aggression by showing that students with high aggression scores were more susceptible to committing suicide [38]. Physiological factors may also play a role in violence among adolescents; an investigation conducted in the USA showed that the level of serotonin in the cerebro-spinal fluid was associated with impulsive aggression as well as suicidal behavior among adolescents [39]. In addition, other research provides additional evidence that bullying is associated with psychological conditions such as depression [40]. Depression itself is a risk factor for suicide among this population [41]. This implies that being bullied is not only implicated in physical fighting behavior but also potentially suicidal behavior.

In this study, the analysis revealed that having understanding and supportive parents reduced the risk of being involved in physical fights among school-attending adolescents in El Salvador. This is consistent with other studies, which revealed that parental support reduces the risk of physical fighting among adolescents [25,26]. A study conducted among African-American youth in the USA concluded that having a close parent–child relationship improved the youth’s ability to select prosocial friends, which was directly related to a decreased involvement in violence [42]. A prior study conducted in El Salvador revealed that Salvadoran adolescents with parents who knew their whereabouts during the weekdays and weekend were less likely to report substance use, aggression, and risky sexual behavior [43].

Moreover, in the current study, loneliness was found to be associated with physical fighting among the study group, after adjusting for age and sex. A study conducted in the USA showed that loneliness plays a role in the development and continuation of violent, antisocial attitudes and behavior [44]. In the USA, on average, there are 1.37 school shootings each week of the school year. Overwhelmingly, these acts are perpetrated by isolated and angry young men [45].

There is a dearth of literature about the association between physical fights and truancy. However, in contrast to our result, a study conducted among Ghanaian adolescents concluded that physical fights are linked to truancy among adolescents [26].

### Strengths and Limitations

The results of the present study, while generally in line with previous literature on the subject matter, must be viewed in light of their limitations. Cross-sectional studies conducted in school-based settings—usually once with no follow-up—only capture the responses of those who showed up on the day of the survey. This means that the responses of those who do not attend school and those absent on the survey day are not represented. Furthermore, because the data are cross-sectional, they do not establish a chain of causality for any of the represented variables. Moreover, single-item measurement is subject to bias, hence the associations between the risk factors and physical fighting may be accordingly over- or under-estimated. Nevertheless, dichotomization of the variables could lead to less precise measurement of those variables. In the context of the present study, it was not possible to either examine the events that led to fighting behavior among the youth or the outcomes of the fights.

## 5. Conclusions

Our results add insight into associations between physical fighting and other risk factors and behaviors among a nationally representative sample of adolescents aged 13–16 years in El Salvador. The study showed which risk factors should be prioritized in dealing with school violence in a Central American lower-middle-income country setting. This study suggests that public health interventions and school-based programs against physical violence among adolescents in schools might benefit from targeting male adolescents by preventing bullying behavior, involving parents, and promoting healthy lifestyles among adolescents in schools. Having an open communication with the students, having a clear definition of bullying in schools, as well as setting clear and enforceable rules and expectations are important for reducing bullying behavior in schools. Nevertheless, organizing positive extracurricular activities, spreading awareness among families, and reducing screen-time for students, and hence promoting healthy lifestyles, are also potentially important in reducing violence in schools.

## Figures and Tables

**Table 1:** Independent variable derivation from Global School-based Health Survey (GSHS) survey data from El Salvador, 2013.

Survey Question	Coding	Variable
Individual-level variables		
How old are you?	12–16 years (coded as continuous)	Age
What is your sex?	Male (1); female (0)	Sex
During the past 30 days, on how many days were you bullied?	0 days (0); 1 or more days (1)	Bullying victimization
During the past 7 days, on how many days were you physically active for a total of at least 60 minutes per day?	4 to 7 days (0); 3 days or less (1)	Physical inactivity
During the past 12 months, how often have you been so worried about something that you could not sleep at night?	Most of the time/always (1); never/rarely/sometimes (0)	Anxiety
During the past 12 months, how often have you felt lonely?	Most of the time/always (1); never/rarely/sometimes (0)	Loneliness
During the past 30 days, on how many days did you miss classes or school without permission?	Zero to 2 days (0); 3 to 10 or more days (1)	Truancy
During the past 12 months, did you make a plan about how you would attempt suicide?	Yes (1); no (0)	Suicide Planning
Social-level variable		
During the past 30 days, how often did you go hungry because there was not enough food in your home?	Most of the time/always (1); never/rarely/sometimes (0)	Food insecurity
During the past 30 days, how often did your parents or guardians understand your problems and worries?	Most of the time/always (0); never/rarely/sometimes (1)	Non-understanding parents
During the past 30 days, how often were most of the students in your school kind and helpful?	Most of the time/always (0); never/rarely/sometimes (1)	Unhelpful peers
How much time do you spend during a typical or usual day sitting and watching television, playing computer games, talking with friends, or doing other sitting activities, such as chatting, playing with cell phone, talking on the phone, using an iPad or tablet?	2 or fewer hours per day (0); 3 or more hours per day (1)	Sedentary lifestyle
How many close friends do you have?	0 = (0); 1 = (1); 2 = (2); 3 or more (3)	Close friends (coded as continuous)

**Table 2:** Distribution of selected factors according to categories of physical fighting among school-attending adolescents in El Salvador, GSHS 2013.

Variable	Not Involved in Physical Fights (n = 1672)	Involved in Physical Fights (n = 238)	*p*-Value
Age (SD)	14.2 (1.1)	14.4 (1.1)	0.158
Sex (male)	49.0	70.9	<0.001
Bullying victimization	20.2	40.5	<0.001
Physical inactivity	76.3	63.6	<0.001
Anxiety	6.0	13.8	<0.001
Loneliness	8.5	12.8	0.028
Truancy	12.0	16.6	0.046
Suicide planning	10.0	22.8	<0.001
Food insecurity	3.2	5.7	0.100
Non-understanding parents	48.0	63.4	<0.001
Unhelpful peers	44.4	58.6	0.002
Sedentary	32.7	46.2	0.002
Close friends (SD)	2.6 (0.8)	2.4 (1.0)	0.047

All variables are expressed as proportions (in %), with the exception of “age” and “close friends”, which are expressed as mean and standard deviation.

**Table 3:** Multivariate analysis of physical fighting among school-attending adolescents in El Salvador, GSHS 2013.

Variable	OR	95% CI (99% CI)	*p*-Value
Age (years)	1.10	0.93–1.30 (0.87–1.38)	0.271
Sex (male)	2.50	1.60–3.85 (1.36–4.52)	<0.001
Bullying victimization	2.82	2.05–3.88 (1.83–4.35)	<0.001
Physical inactivity	0.60	0.46–0.79 (0.41–0.87)	0.001
Anxiety	2.96	1.88–4.67 (1.59–5.51)	<0.001
Loneliness	2.10	1.37–3.23 (1.17–3.78)	0.002
Truancy	1.30	0.88–1.91 (0.77–2.20)	0.174
Suicide planning	3.40	2.10–5.49 (1.77–6.53)	<0.001
Food insecurity	1.62	0.71–3.70 (0.53–5.00)	0.236
Non-understanding parents	1.97	1.54–2.52 (1.41–2.76)	<0.001
Unhelpful peers	1.71	1.22–2.39 (1.09–2.69)	0.003
Sedentary lifestyle	1.91	1.30–2.80 (1.14–3.21)	0.002
Close friends	0.89	0.80–0.99 (0.77–1.03)	0.038

OR—odds ratio. 95% CI and 99% CI—95% and 99% confidence interval. All estimates are adjusted for age and sex; sex and age were adjusted for each other.

**Table 4:** Outcomes of the multivariate analysis of variables associated with physical fighting attempts among school-attending adolescents in El Salvador, GSHS 2013.

Variable	Adjusted Odds Ratio	95% CI (99% CI)	p-Value
Sex (male)	3.55	2.11–6.00 (1.75–7.19)	<0.001
Bullying victimization	2.16	1.44–3.24 (1.24–3.75)	<0.001
Physical inactivity	0.61	0.46–0.80 (0.42–0.88)	0.001
Anxiety	1.29	0.64–2.60 (0.50–3.35)	0.453
Loneliness	1.07	0.59–1.95 (0.47–2.42)	0.816
Truancy	1.07	0.69–1.66 (0.59–1.95)	0.758
Suicide planning	2.28	1.46–3.56 (1.24–4.18)	0.001
Non-understanding parents	1.45	1.06–1.98 (0.94–2.22)	0.023
Unhelpful peers	1.39	0.93–2.09 (0.80–2.42)	0.103
Sedentary lifestyle	1.54	1.05–2.27 (0.91–2.61)	0.030
Close friends	0.99	0.80–1.23 (0.74–1.33)	0.956

Only those factors found to be statistically significant in the bivariate analysis were used in this model. OR—odds ratio. 95% CI and 99% CI—95% and 99% confidence interval. All estimates are adjusted for all variables listed in the table.
